# High resolution depth profile scanning of plankton organisms—VERTILICE

**DOI:** 10.1016/j.mex.2024.102784

**Published:** 2024-05-29

**Authors:** Bjarne Kvæstad, Andreas Hagemann, Frode Leirvik, Birger Venås

**Affiliations:** a SINTEF Ocean, Fisheries and New Biomarine Industry, Brattørkaia 17C, 7010 Trondheim, Norway; b SINTEF Ocean, Climate and Environment, Brattørkaia 17C, 7010 Trondheim, Norway; c SINTEF Ocean, Aquaculture, Brattørkaia 17C, 7010 Trondheim, Norway

**Keywords:** Fully convolution network, Machine learning, Imaging, Spectrometer, Sea-lice, Automation, Classification, Image processing, Depth profile, VERTILICE

## Abstract

Knowledge of the vertical migration pattern of sea lice (*Lepeophtheirus salmonis*) copepodites is necessary for designing efficient measures to prevent lice infestations on farmed Atlantic salmon (*Salmo Salar*) in sea-cages. However, data can be challenging to acquire at a large scale under realistic circumstances without interfering with the natural behavior of the specimen. A mesocosm platform was built to help acquire this data consisting of a sensor package in an underwater housing being pulled up and down along a 11-meter-long transparent tube containing planktonic organisms while collecting image-, temperature- and spectrometer data. The platform was placed at a salmon farm and the acrylic tube was filled with *L. salmonis* copepodites and was pre-programmed to run a profile scan twice per hour for four consecutive days. Using a fully convolutional neural network, the copepodites were automatically counted – creating a depth profile of detected lice and measured light specter. The final results showed a diurnal migration pattern throughout the test period.

• Capable of acquiring vertical density profiles of any aquatic species between 0,5 – 10 mm down to 11 m below the surface.
• Fully automated and can be left unintended for weeks while collecting data.

## Graphical abstract

**Figure:**
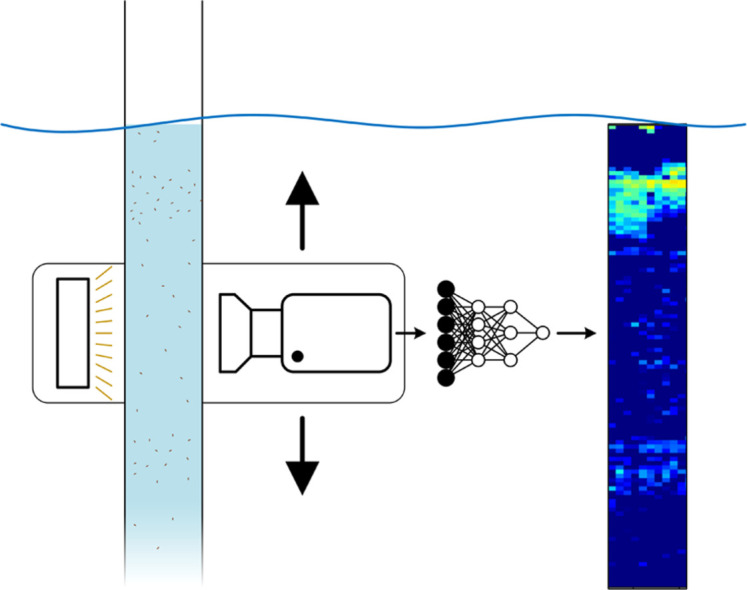

Specifications table

**Table:** 

Subject area:	Environmental Science
More specific subject area:	Behavioral study
Name of your method:	VERTILICE
Name and reference of original method:	n/a
Resource availability:	https://doi.org/10.5281/zenodo.7418314

## Method details

There are many different approaches for profiling particles in the water column by using camera technology in combination with machine learning [[1], [2], [3]]. These systems can be used for profiling free-swimming organisms and other particles in the ocean, however, they are not the right tool to use if the aim is to study the vertical displacement of a population of zooplankton in relation to natural light cycles without hydrodynamic interference.

The development of VERTILICE platform started in 2019 by running small scale experiments in the laboratory to test the concept of automated counting of copepodids in an acrylic tube. Using this knowledge a large-scale platform was built and tested in January 2020. A three-day long field experiment where the rig was installed at a local dock was conducted, yielding meaningful results, a diurnal migratory pattern was observed, though the trend was not clear enough for publishing. The platform presented in this article is the culmination of this iterative development and testing conducted between 2019 and 2022.

VERTILICE provides a platform for studying the vertical migration pattern or distribution of plankton in the 0.5–10 mm size range inside a vertical 11-meter-long acrylic tube in the water column. The platform is fully automated and can be programmed to run regular scans to acquire image data with corresponding environmental data (light, temperature, depth) from 0 to 11 m below the surface. The platform and method were demonstrated and validated in a mesocosm setup where the diurnal vertical migration of sea lice (*Lepeophtheirus salmonis*) copepodites during a 4-day trial was monitored.

### Data acquisition

**Fig. 1:**
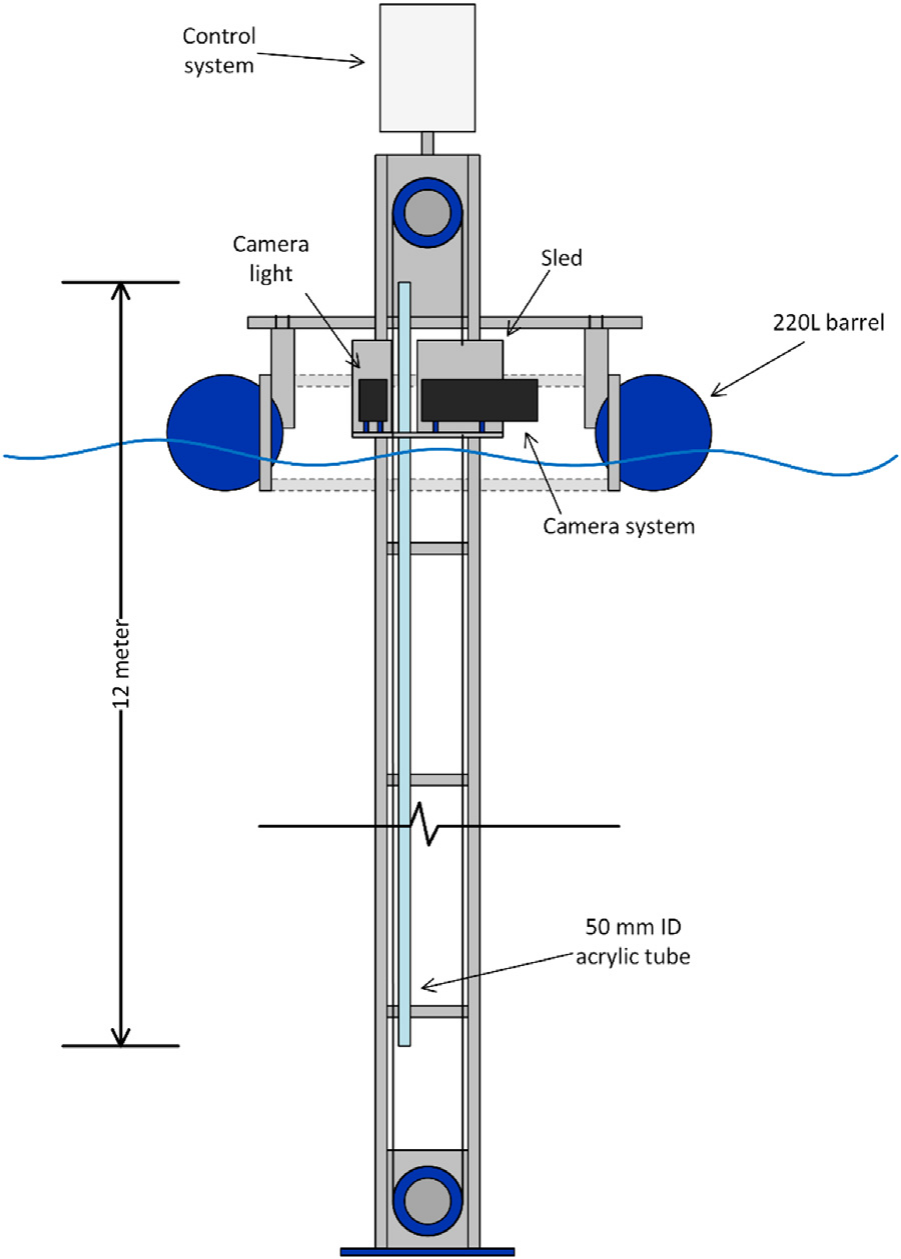
Schematic illustration of the VERTILICE rigs’ positioning in the sea. (For interpretation of the references to color in this figure legend, the reader is referred to the web version of this article.)

A motorized sled structure with a camera system was made to image 5 cm sections (∼0.1 L volume) of a 12-meter-long acrylic tube (5 cm inner diameter) filled with sea water and *L. salmonis* copepodites. The acrylic tube was mounted on an aluminum frame consisting of three modules, (i) top module with control system and pulley system, (ii) middle rail module and (iii) end rail module, measuring 14 m long, where the tube spanned 11 m below the water surface. The tube consisted of 2-meter-sections spliced together with a 5 cm rubber sleeve and hose clamps. This modular design makes VERTILICE customizable in terms of length, as the system can be assembled to span 11 or 5 m under the water surface by omitting the middle rail module. In theory the system can be extended to go even deeper by adding more modules, but this is yet to be tested. A control system, controlling the sled and acquiring data from the camera system was mounted on top. The aluminum frame was mounted on a buoy made from four 220 L high-density polyethylene (HDPE) barrels, allowing the frame to move freely along the pitch- and roll axis during high waves (Fig. 1, Appendix A and B).

**Fig. 2:**
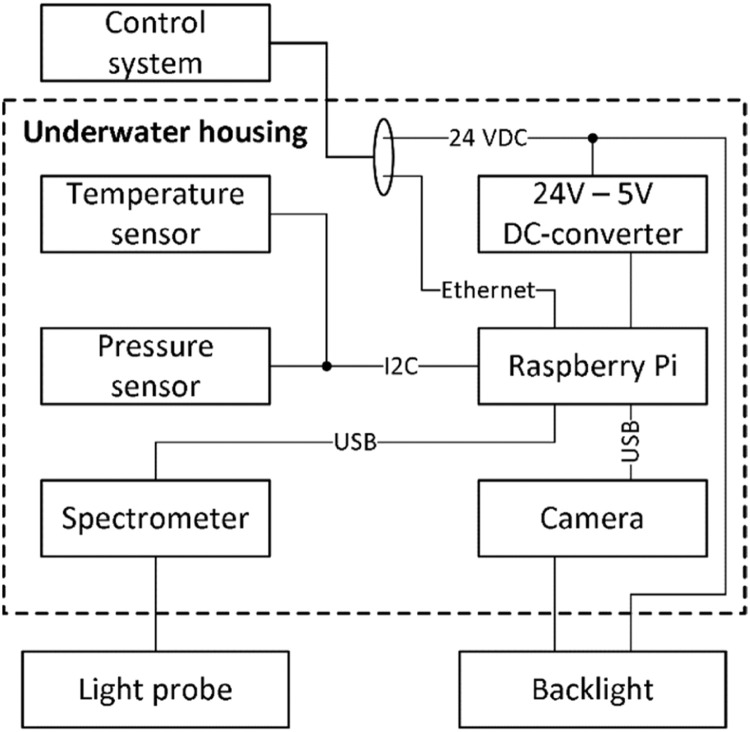
Block diagram of the components in the underwater housing and how they are connected.

The camera system consisted of a camera (Alvium 1800 U-501 NIR, Allied Vision, USA) with a telecentric lens (0.125 × 2/3″ GoldTL™ Telecentric Lens, Edmund Optics, USA) and an infrared backlight (CB-series 3 × 3″ 880 nm, Advanced Illumination, USA) placed on the opposite side of the tube for bright field imaging. The camera setup along with the following sensors were built into a watertight enclosure (Acryllic 4″ x 50 cm, BlueRobotics, USA) and connected as shown in Fig. 2.

- Temperature sensor (Celsius Fast-Response, ±0.1 °C Temperature Sensor (I2C), BlueRobotics, USA))
- Pressure sensor for depth measurement (Bar30 High-Resolution 300 m Depth/Pressure Sensor, BlueRobotics, USA)
- Spectrometer (FLAME-S-UV–VIS-ES, 350 – 1000 nm, Ocean Optics, USA) with a spherical light collector probe (A-COL-ESCALAR, Hobi Instrument Services, USA)

Software written in Python 3.6 running on a Raspberry Pi 4 Model B inside the underwater housing was used to acquire and synchronize the data streams from the camera, -temperature, -pressure and -spectrometer and stored to a hard drive in the top-side control system. The sample-rate of the spectrometer was varying between 0.1 Hz and 1 Hz due to the variation of light intensity conditions (day/night), a P-regulator was implemented to automatically set the integrating (exposure) time so that the max photon count would be 50,000 (of 64,000 max) in all light conditions. The image sample-rate was set to 0.5 Hz, giving an image profile overlap of 15 mm . The remaining sensor data was timestamped with approximately 1 Hz. The components were powered by 24VDC via an underwater cable from the top-side control system along with an ethernet connection for communication between the control system and camera system.

**Fig. 3:**
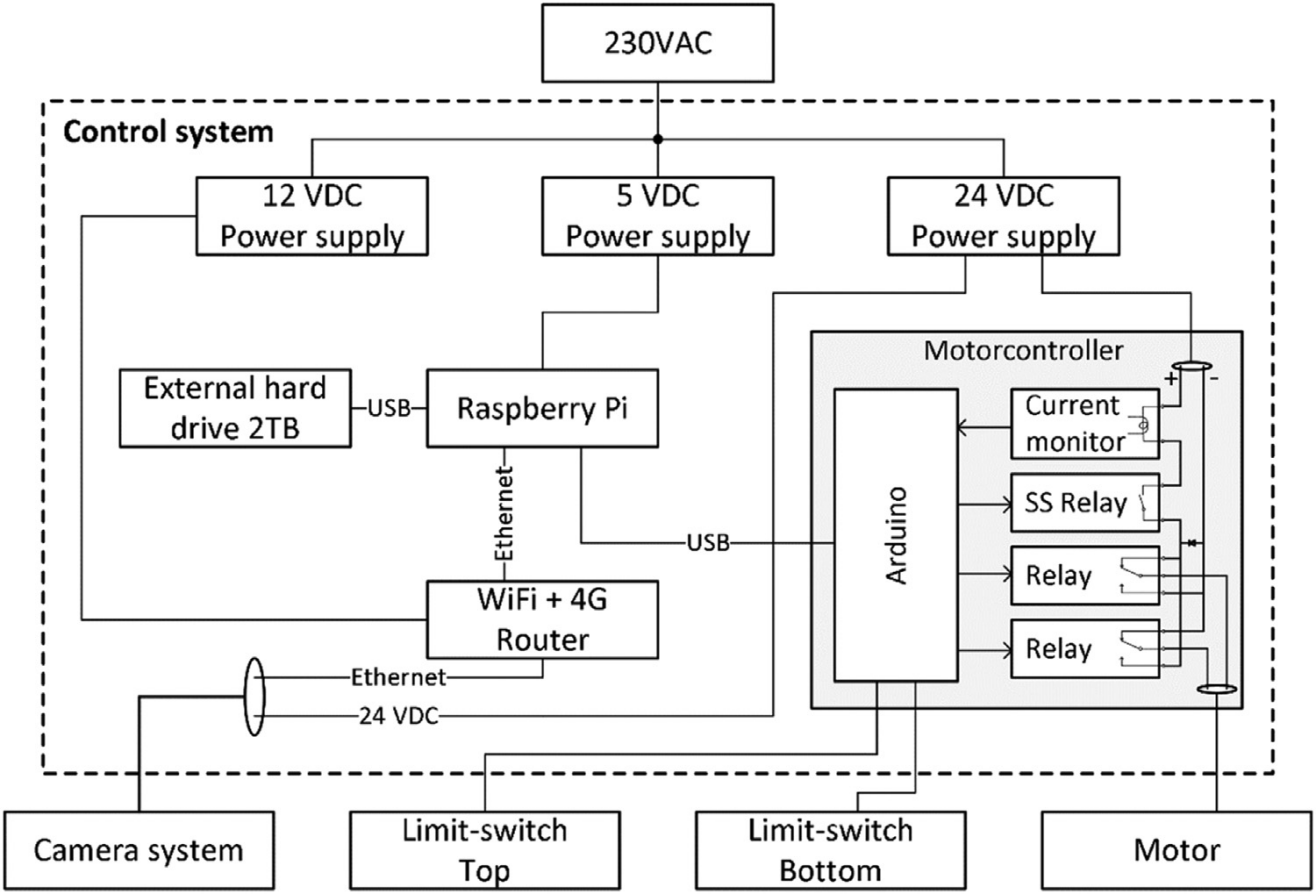
Block diagram of the components in topside control system.

The control system consisted of a Raspberry Pi 3 Model B, a hard drive (2000 GB, T7, Samsung, South Korea), a 4 G modem (RUTX11 LTE CAT6, Teltonika, Lithuania) and a custom-made DC motor controller for running the sled motor (Fig. 3).

The control system was programmed to run a pre-determined schedule of two scans per hour. When a scan was initiated, the control system started the sled along with sending a signal to the camera system. This started the image- and sensor data acquisition, this was stopped after the sled completed one cycle (down and up). A timestamped folder was automatically generated on the hard drive in the control system for storage of image-, temperature-, depth- and spectrometer data. The sled speed corresponded to 30 s per meter, which resulted in a travel time of ∼12 min to finish one scan (Appendix C).

The sled was powered by a 24 V DC motor (209 W 10 Nm 686 RPM, Crouzet, France) placed topside under the control system junction box. The bi-directional motor was connected to a reel which pulled a wire (and the sled) up or down. Using two limit-switches the motor controller would automatically reverse the motor direction when the sled was at the bottom, and stop when the sled was in parked position, ∼0.5 m above the water surface. The controller was built based on an Arduino Uno and a solid-state relay, featuring control via serial communication over USB (from the Raspberry Pi), overcurrent protection and motor speed- and direction adjustment. The overcurrent protection served as redundancy in cases where a faulty limit switch could cause the motor to burn out or break the pulley system.

### Classification and counting

Using Python 3.6 and PyTorch 1.10, a custom Fully Convolutional Network - FCN [6] was developed to detect the copepodites in the images from the sled, the neural net architecture is presented in Table 1.

**Table 1:** Neural network architecture.

Layer	Type	Input channels	Output channels	Kernel size	Activation type
Input layer	Conv2d	1	8	3 × 3	ReLU
Hidden layer 1	Conv2d	8	16	3 × 3	ReLU
Hidden layer 2	Max_pool2d			2 × 2	
Hidden layer 3	Conv2d	16	32	3 × 3	ReLU
Hidden layer 4	Max_pool2d			2 × 2	
Hidden layer 5	Conv2d	32	64	3 × 3	ReLU
Hidden layer 6	Conv2d	64	512	12 × 12	ReLU
Output layer	Conv2d	512	1	1 × 1	Sigmoid

**Fig. 4:**
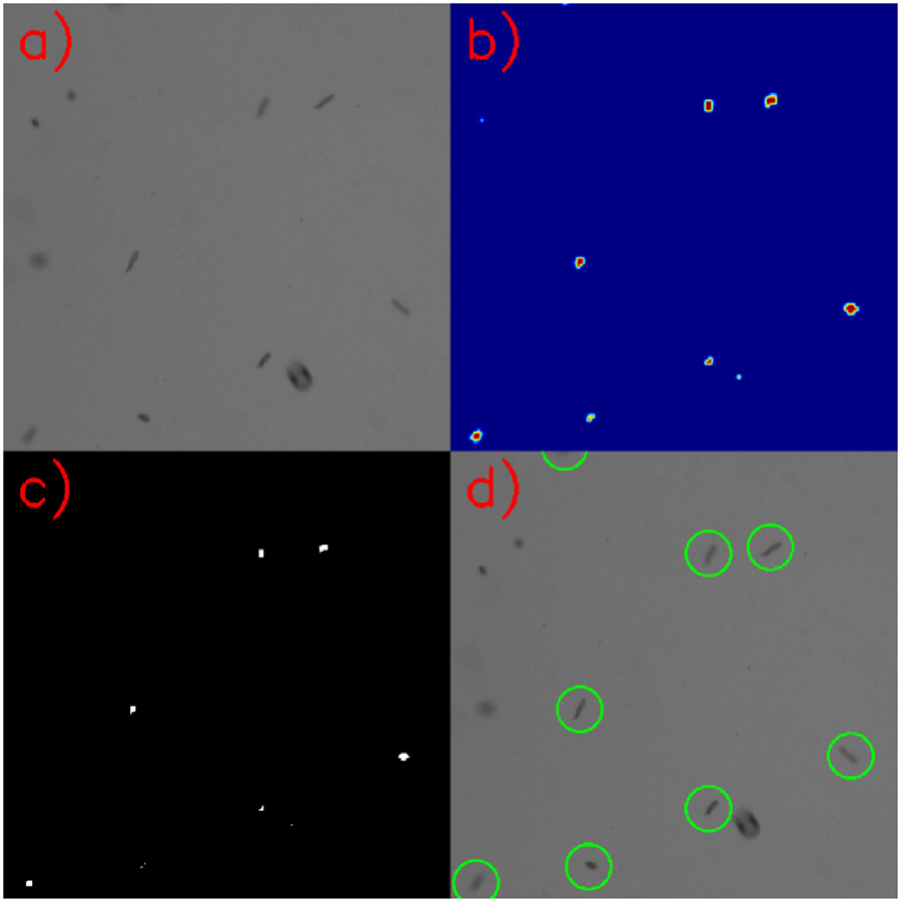
Illustration of the processing flow of a raw image from the sled (a), and the output from the neural network (b) where blue pixels are classified as background or other particles and red pixels are classified as a copepodite, where the threshold of 0.8 was applied (c) and “findContours”-function to detect and count copepodites (d). (For interpretation of the references to color in this figure legend, the reader is referred to the web version of this article.)

The FCN architecture consists of only convolutional- and max pooling layers, meaning that the output of the neural network will automatically scale to the input. The input is a raw image of *n* x *m* pixels, and output is an image of (*n*-32) x (*m*-32) pixels. Each pixel in the output image represents a confidence-value between zero and one, where “zero” is classified as background and “one” is classified as a copepodite. A threshold of 0.80 was set on the output image to filter out classifications with low confidence. Using the “findContours”-function from OpenCV, based on Suzuki et al. [8], the number of copepodites in one image were detected and counted (Fig. 4).

**Fig. 5:**
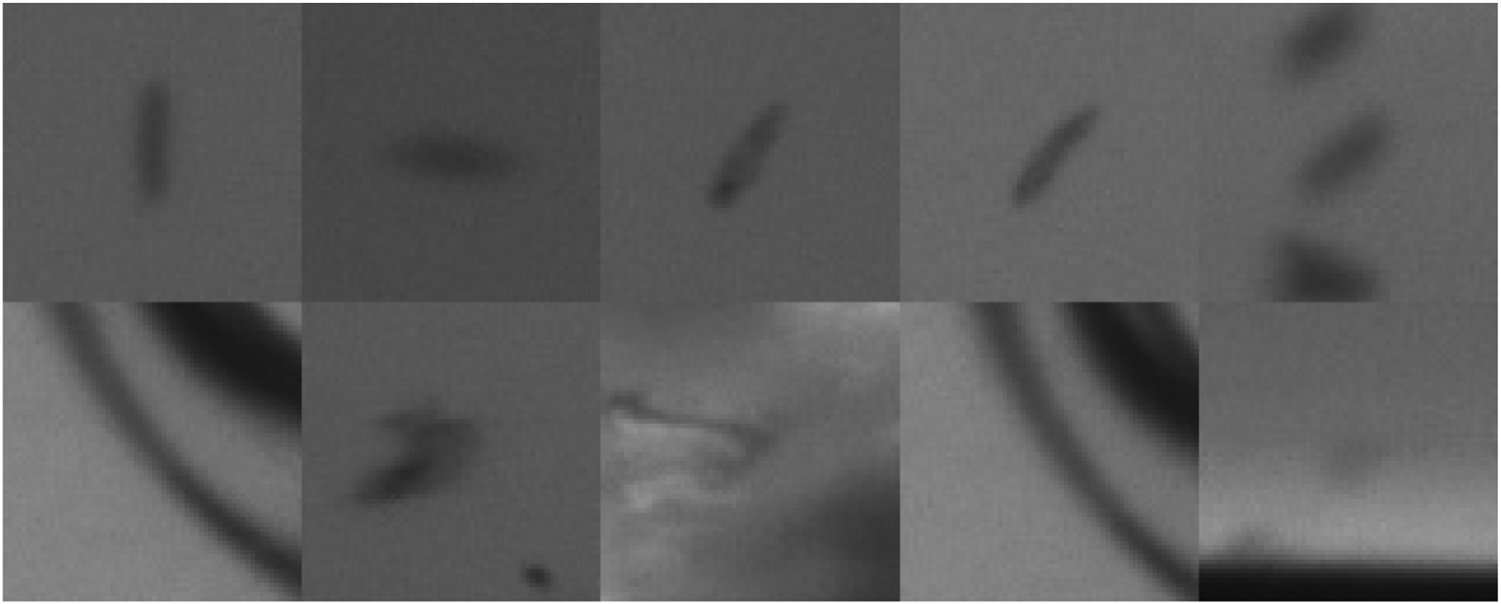
Samples of training data containing copepodites (top row) and background or other particles (bottom row).

Due to the nature of the FCN architecture, it is not needed to annotate whole images, but only parts of it. Due to the scaling of this specific neural network architecture, an image of 64 × 64 pixels will give an output of one value (1 × 1 pixels, ranging from zero to one), so when annotating, small thumbnails can be extracted and annotated with either “1” for a copepodite or “0” for background depending on what is in the center of the extracted thumbnail, see Fig. 5 for examples.

The neural network was trained on 1846 thumbnails (1022 of copepodites and 824 of background/other), where 10 % of the dataset was used for validation. The training data was selected using a custom annotation tool written in Python 3.6. This annotation tool will first process the image using the FCN with the newest model weights. This way, when annotating images, an insight is given into what the model had learned, and what kind of training data it needs more of. The process of data annotation and training was repeated eight times. To expand the training set further, image augmentation was applied. Images were randomly flipped vertically and horizontally and randomly rotated between 0 and 359°. These augmentation techniques were selected because the outcome of the augmentations reflects a realistic orientation of the copepodid. A dropout of 60 % was also applied between layer 6 and the output layer to improve generalization and prevent overfitting. The validation loss plateaued around epoch 455, giving an accuracy of 95.6 % when tested on the validation data set.

### Sensor data handling

The spectrometer and the light collector probe were calibrated for water-immersed conditions by Hobi Instrument Services in July 2017, giving a calibration file for converting the light specter from photon counts per wavelength to Joules per wavelength ($J_{\lambda }$). The spectrometer data was converted to light intensity (I) in $\mu Mol/m^{2}s^{2}$ with the following formula [9].

$$I=\;\frac{\sum_{\lambda =350}^{1000}\frac{J_{\lambda }\cdot A\cdot t}{E_{\lambda }\cdot N_{A}}}{t\cdot A\cdot 10^{-4}}$$

Where A is the area of the light collector probe (1 cm^2^), t is the integration time of the spectrometer, $N_{A}$ is Avagadro's constant and $E_{\lambda }$ is amount of energy per photon, calculated with the following formula:

$$E_{\lambda }=\frac{h\cdot c}{L_{\lambda }\cdot 10^{-9}}$$

Where h is the Planck constant, c is the speed of light and $L_{\lambda }$ is the wavelengths measured by the spectrometer in nano meters.

The detected copepodites-, temperature-, spectrometer- and pressure data was synchronized using the timestamps so that the data could be linked with the depth measurement to get vertical profiles in the water column. As the integration time of the spectrometer could be as high as 10 s, the sampling rate of this sensor would drop to 0.1 Hz when there was little light available. In order to synchronize the data sampling times, all sensor data was loaded into a Pandas (v1.15) data frame one row per data point, using the “pandas.interpolate” function with method=‘time’ parameter, the data was interpolated in regard of the time. To compensate for the change in atmospheric pressure, an average of the first ten samples per scan (when the sled was parked above the water surface) was used to calibrate the pressure sensor.

## Method validation

VERTILICE was transported in modules and assembled at the land facility of SalMar Farming AS at Korsneset, Norway (63.141914, 8.220516). The rig was transported by crane boat to a salmon sea-cage where VERTILICE got lifted into the net-pen using a crane. VERTILICE was secured by six ropes, four ropes securing the rig in the middle of the pen, and two ropes keeping the frame horizontal (*cf*. Appendix A). When deployed the overcurrent protection unit was adjusted and tested by manually holding back the sled until the overcurrent protection unit triggered and the motor stopped.

The acrylic tube was then filled with 876 copepodites and left to run from the May 3rd¬ to May 7th, 2022, with a two-hour break on May 4th to fix a leak in the underwater housing. The aim of this experiment was to study the diel vertical migration pattern of sea lice (*L. salmonis*) copepodites. The results of this experiment is presented in full in Hagemann et al. [4], After the experiment, the data was copied from the external hard drive and processed with the methods explained in this article. For each scan, the copepodite-, light- and temperature measurements were aggregated into 10 cm vertical depth bins and divided by the number of data measurements per 10-cm bin. For the copepodite data, an average density profile was calculated by summing each profile scan along the time axis (*n*^th^ bin with *n*^th^ bin) and then divide the sum by the number of scans. This average density profile was then subtracted from every scan in the time series, and bins with a negative number were set to zero. This was done to isolate the vertical movement of the copepodids and mitigate scratches and particles stuck to the acrylic tube misclassified as copepodids by the FCN.

**Fig. 6:**
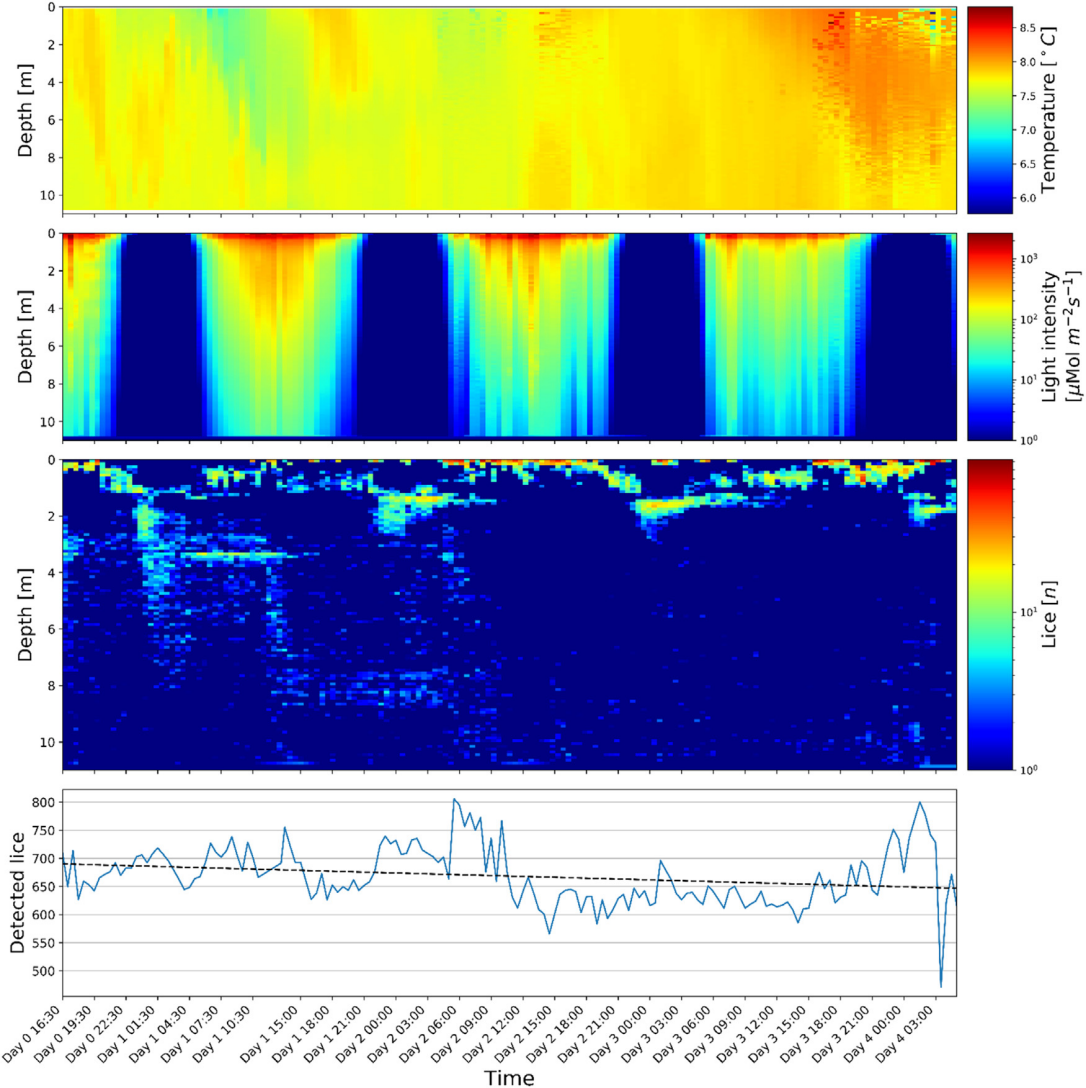
From top to bottom: Time series (four days) of depth profiles: temperature [ °C], light intensity [μMol⁄(m^2 s^2)] and detected copepodites [#]. Bottom plot shows total number of detected copepodites per scan. (For interpretation of the references to color in this figure legend, the reader is referred to the web version of this article.)

During the experiment, a total of 179 scans were executed (two scans per hour), where a total of 14 scans were dropped due to unwanted particles in the acrylic tube (4) and incomplete scans due to delayed camera initialization and faulty limit switch (10), giving a total of 165 complete scans for this experiment. Out of the initial 876 copepodites an average of 790 were detected in the first scans, with a decreasing rate of detections of approximately 12 copepodites per 24 h. Both developmental time and survival for planktonic *L. salmonis* ontogenetic stages are strongly influenced by salinity and temperature [5]. The duration of the infective copepodite stage was found by Samsing et al. [7] to be approximately 13 - 14 days at 7 - 10 °C. The copepodites used for this experiment were up to 6 days old, hence, this observed decrease in detections was most likely not caused by senescence but could still, however, have been caused by mortality during the experimental period. The 5cm-long sleeve used to splice the 2-meter-sections of the acrylic tubes introduced a blind-spot for the copepodite counting, which can be seen in the third subplot in Fig. 6 where the splices are located around 1-, 3-, 5-, 7- and 9-meter depth. The blind spots cover only ∼ 2 % of the total area and does not affect the results which indicate a clear trend of a diurnal pattern. There are some variations in the lice counts (500–800 detections per scan; Fig. 6 subplot 4). This might be caused by lice being oriented parallel to the camera making them appear as a round dot, hence not being classified as a copepodite. Furthermore, the lens focal length is slightly smaller than the acrylic tube diameter rendering a small area along innermost- and outermost of the tube walls out of focus.

The overall lice detections could be improved by using a smaller diameter tube to get the entire volume in focus. Imaging the tube from two angles using two cameras could also be a solution regarding lice orientation and focus area, though it would complicate the setup a lot. Regardless, the total lice counts are relative stable over the 165 scans and from the data, illustrating a repetitive trend over the full duration of the experiment, we see that the VERTILICE platform performs as intended.

## Ethics statement

We confirm that any aspect of the work covered in this manuscript that has involved experimental animals has been conducted with the ethical approval of relevant bodies.

## CRediT authorship contribution statement

**Bjarne Kvæstad:** Methodology, Software, Data curation, Writing – original draft, Visualization. **Andreas Hagemann:** Conceptualization, Investigation, Resources, Writing – review & editing, Supervision, Funding acquisition. **Frode Leirvik:** Methodology. **Birger Venås:** Conceptualization, Methodology, Writing – review & editing, Project administration.

## Declaration of competing interest

The authors declare that they have no known competing financial interests or personal relationships that could have appeared to influence the work reported in this paper.

## Data Availability

Data will be made available on request.

## Appendix Supplementary materials

Appendix A – Images of the VERTILICE platform Image, application 1

Appendix B – Video of the VERTILICE platform in the water Image, video 2

Appendix C – Video of the camera system on its way up Image, video 3
