# Supplementary figures and images for: High resolution depth profile scanning of plankton organisms—VERTILICE
Source: MethodsX. 2024 May 29;12:102784. doi: 10.1016/j.mex.2024.102784 (PMC11176768; doi:10.1016/j.mex.2024.102784)

Appendix A – Images


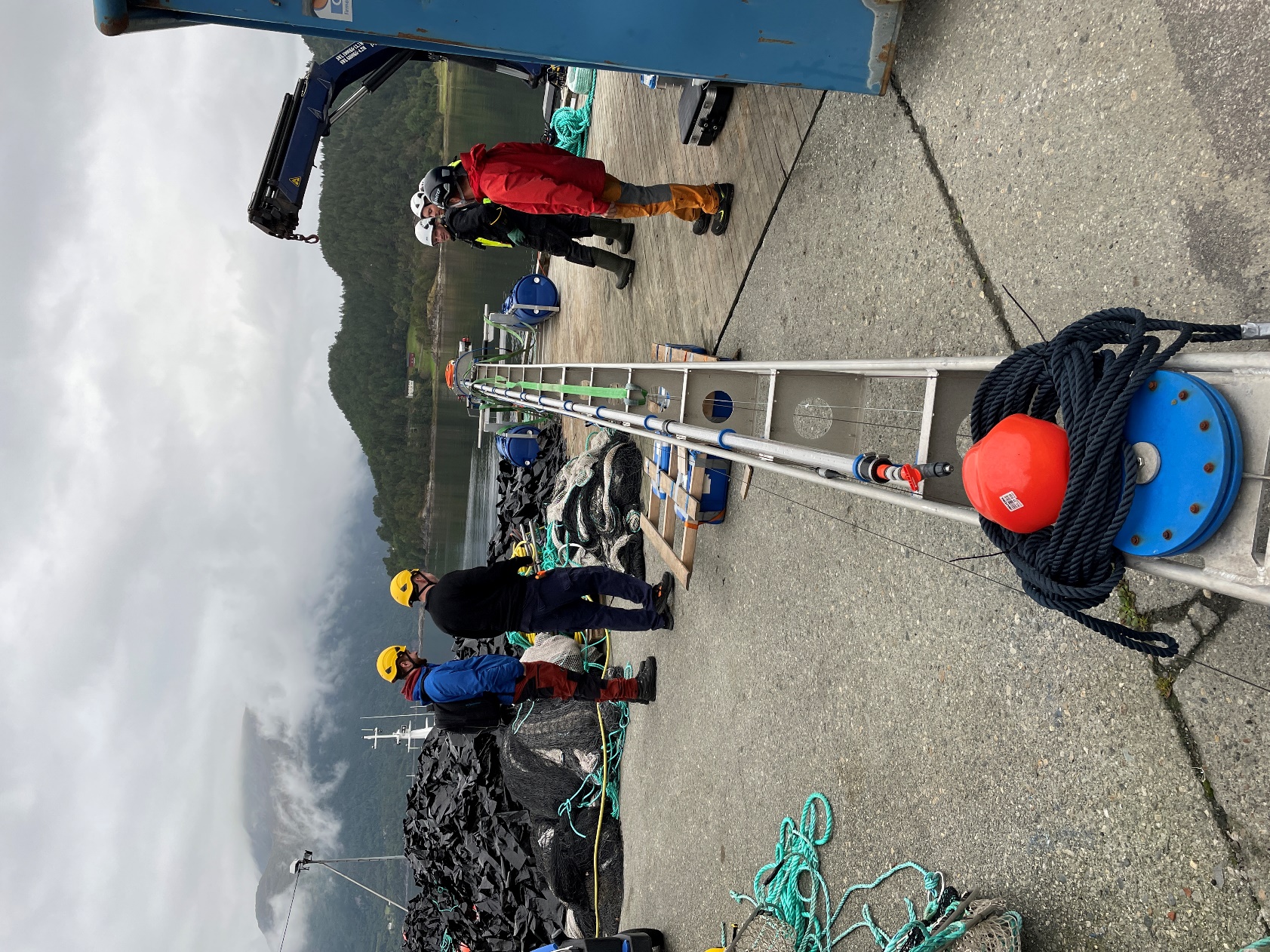


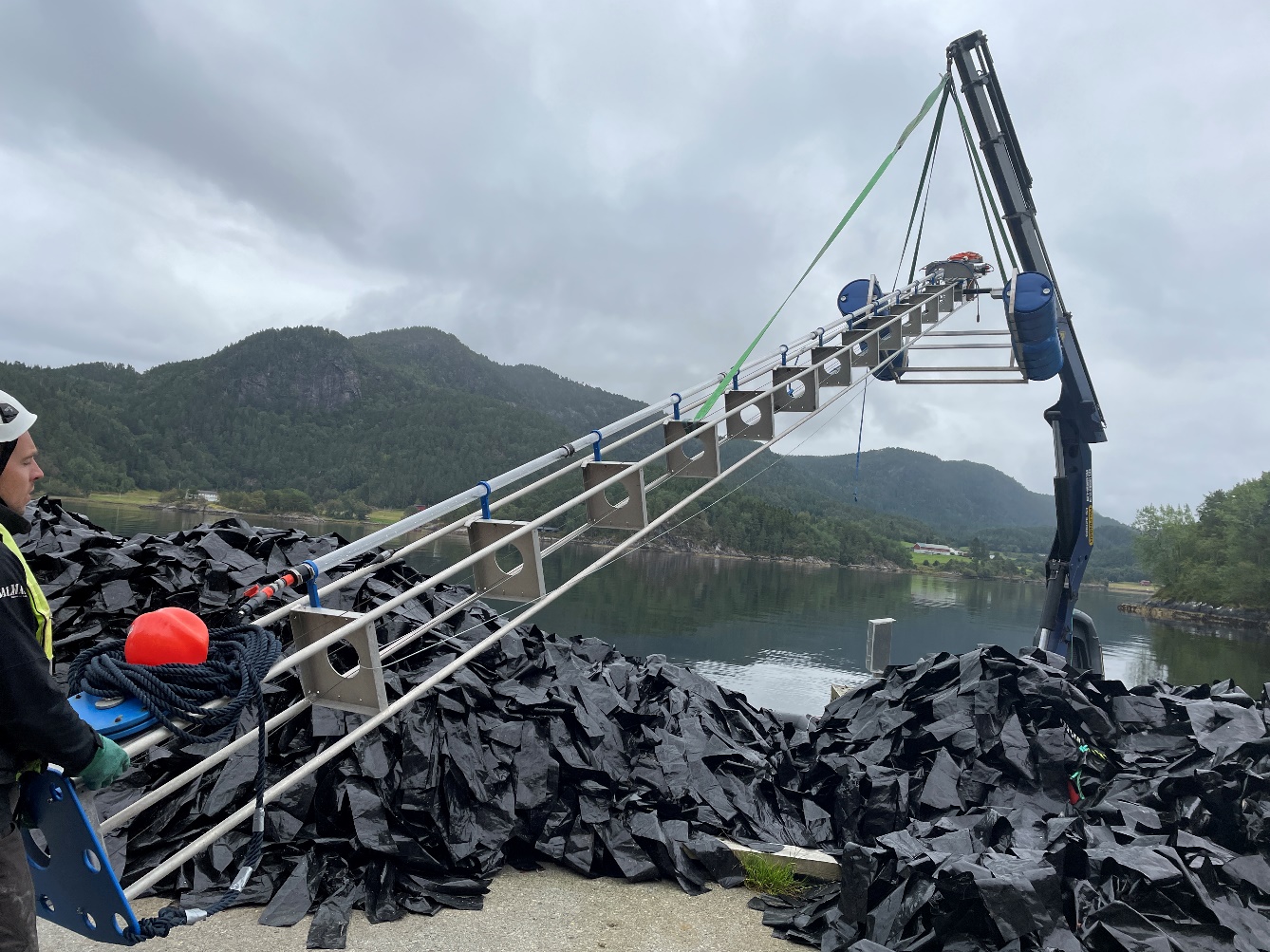

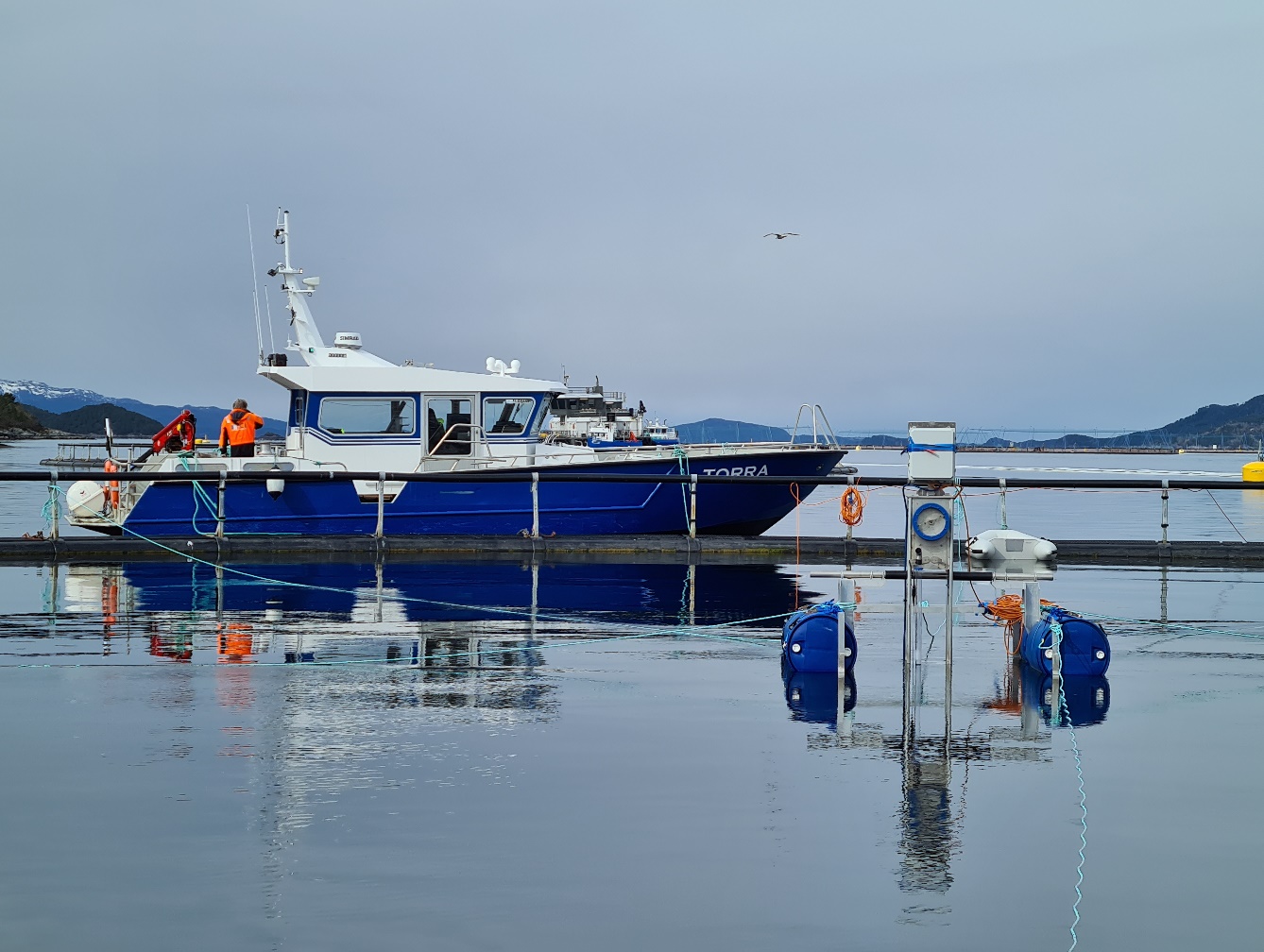


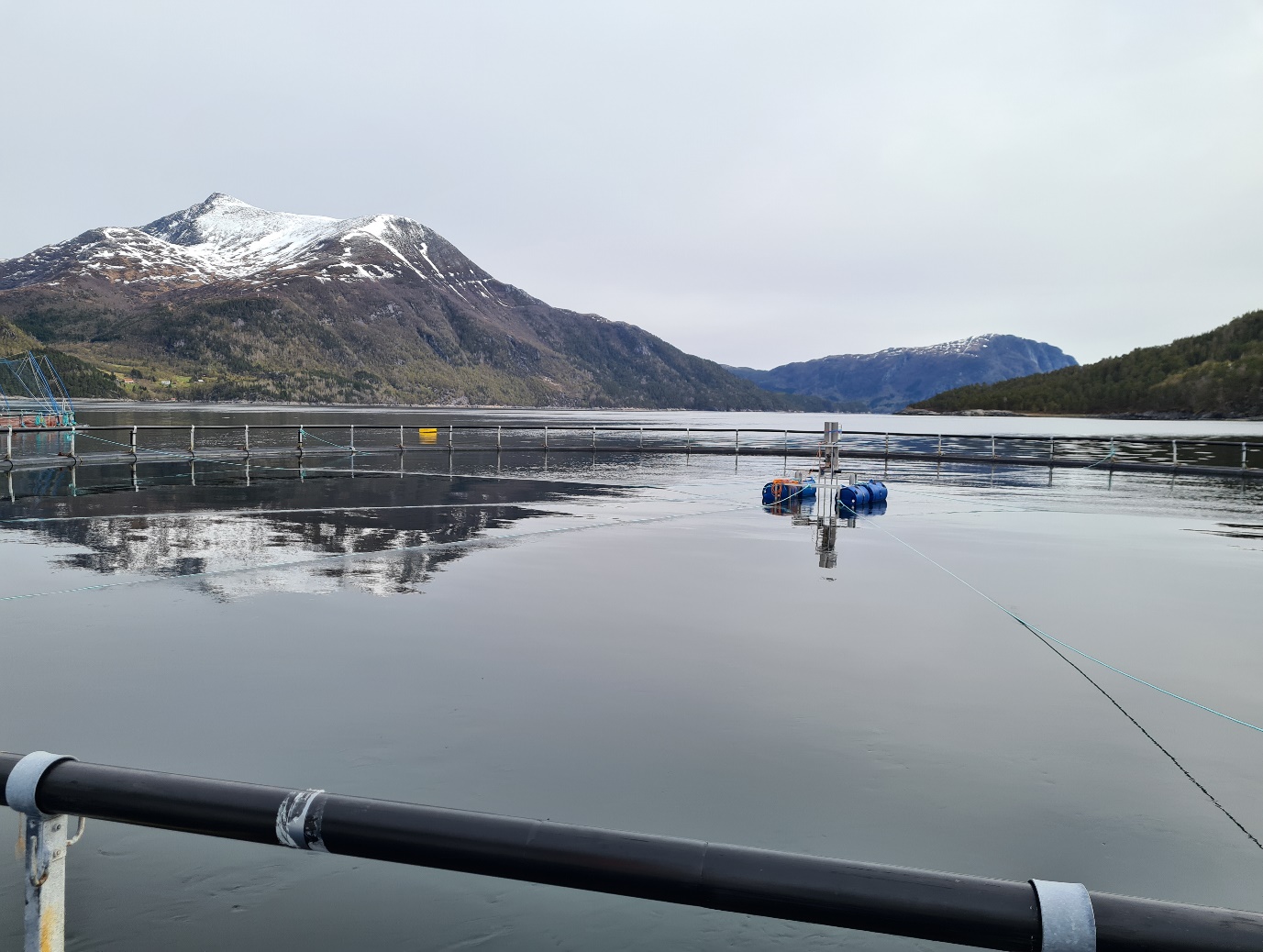

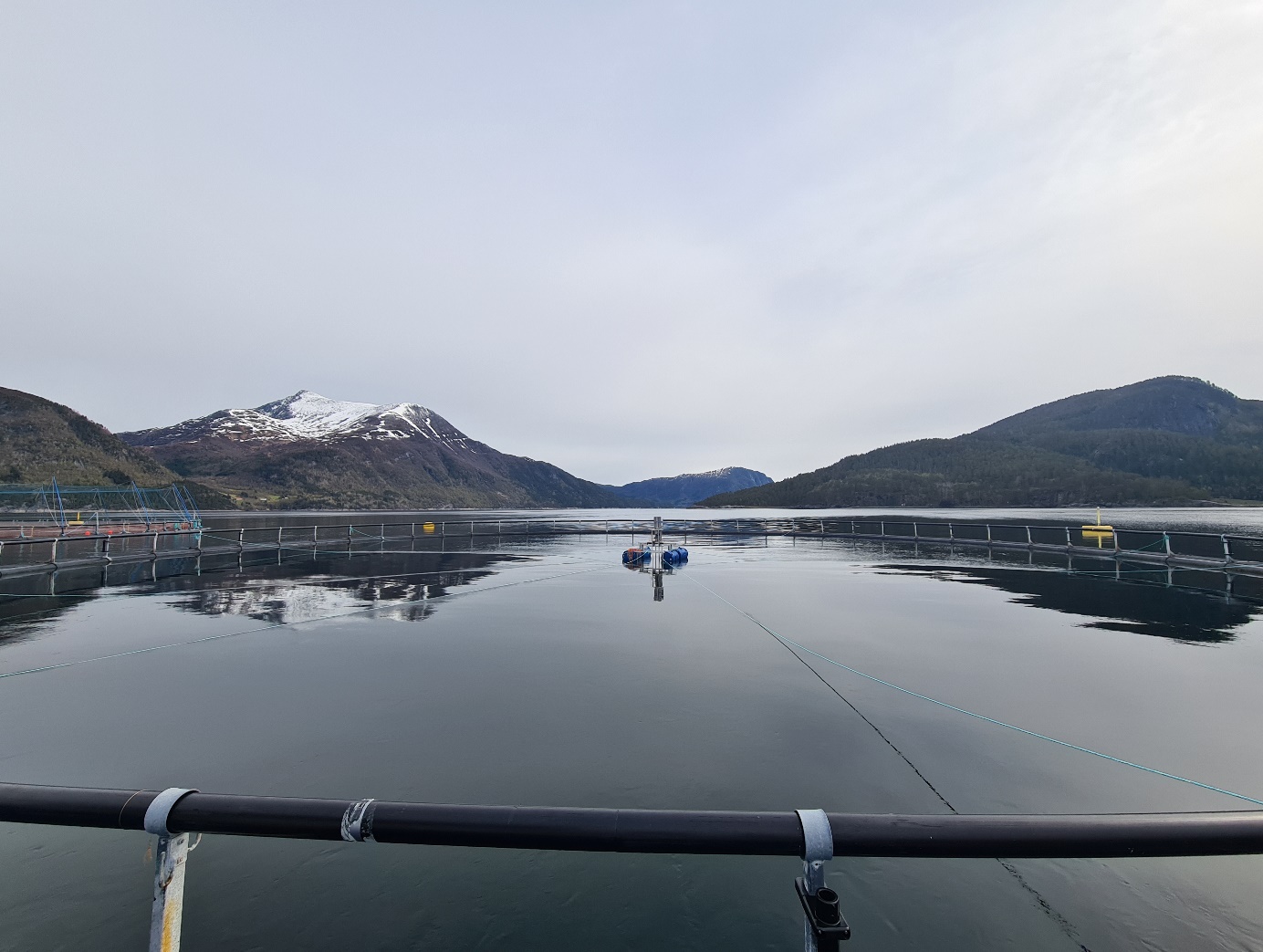

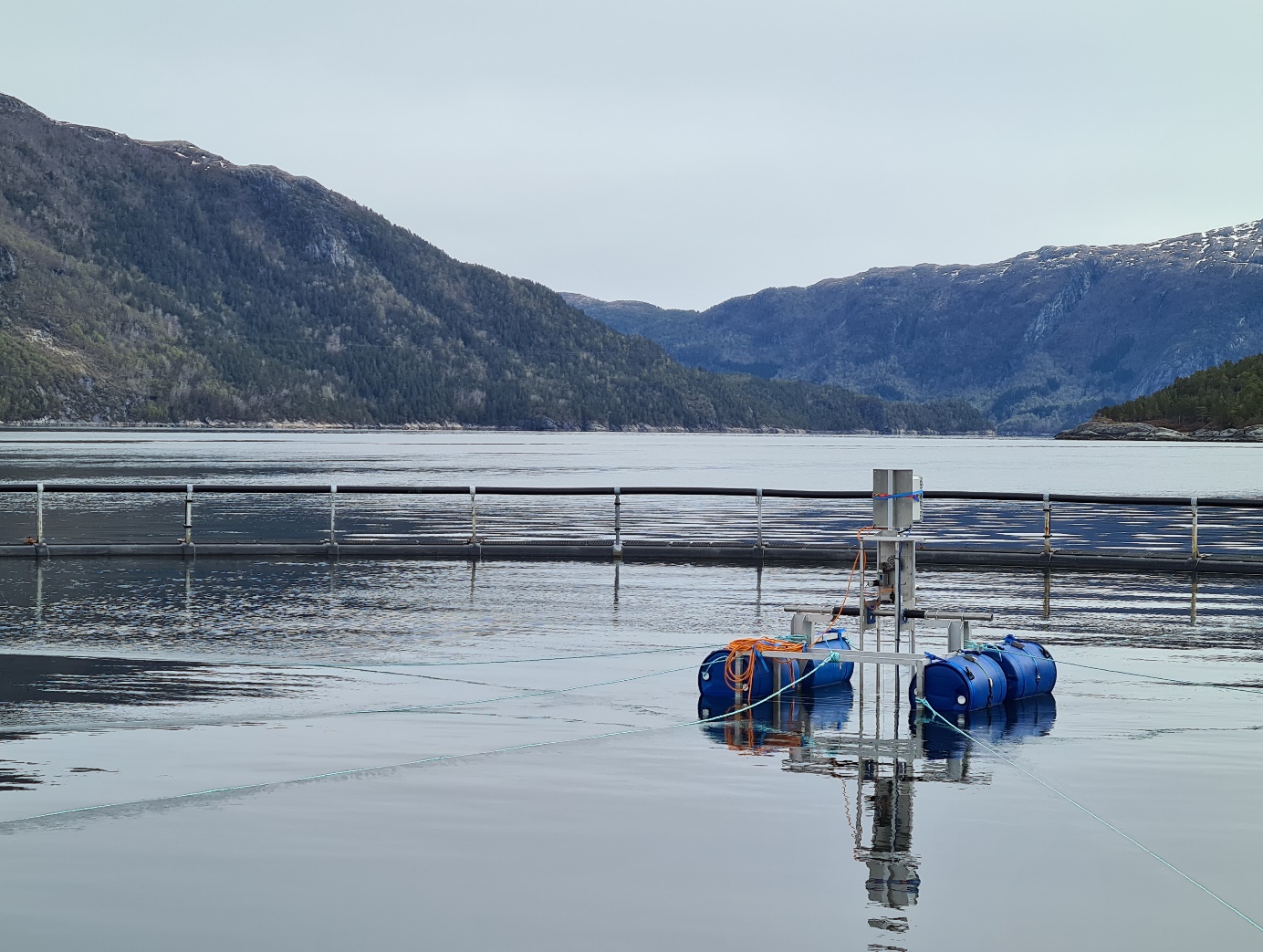

Supplement: Supplementary file 1 — Appendix A – Images of the VERTILICE platform [file mmc1.docx]
